# A new dynamic nomogram for predicting the risk of severe *Mycoplasma* *pneumoniae* pneumonia in children

**DOI:** 10.1038/s41598-024-58784-3

**Published:** 2024-04-09

**Authors:** Xue Zhang, Ruiyang Sun, Wanyu Jia, Peng Li, Chunlan Song

**Affiliations:** Henan Province Engineering Research Center of Diagnosis and Treatment of Pediatric Infection and Critical Care, Children’s Hospital Affiliated to Zhengzhou University, Zhengzhou, 450052 Henan China

**Keywords:** Risk factors, Signs and symptoms

## Abstract

*Mycoplasma pneumoniae* pneumonia (MPP) is usually mild and self-limiting, but still about 12% of them will progress to severe *Mycoplasma pneumoniae* pneumonia (SMPP), which have poor survival rates and often require intensive medical resource utilization. We retrospectively collected clinical data from 526 children with MPP admitted to the Children’s Hospital Affiliated to Zhengzhou University from June 2018 to February 2023 and randomly divided the data into a training cohort and a validation cohort at a ratio of 4:1. Univariate and multivariate logistic regressions were used to identify independent risk factors for SMPP. Age, AGR, NLR, CRP, ESR, MPV, coinfection, pleural effusion, primary disease, fever days ≥ 7 and wheeze are independent risk factors for SMPP in children. Then, we built an online dynamic nomogram (https://ertongyiyuanliexiantu.shinyapps.io/SMPP/) based on the 11 independent risk factors. The C-index, ROC curve, DCA curve and calibration curve were used to assess the performance of the nomogram, which all showed that the dynamic nomogram has excellent clinical value. Based on age, AGR, NLR, CRP, ESR, MPV, coinfection, pleural effusion, primary disease, fever days ≥ 7 and wheeze, the first dynamic nomogram for accurately predicting SMPP was successfully established.

## Introduction

*Mycoplasma pneumoniae* pneumonia (MPP) is the predominant community-acquired pneumonia in children aged 5 years and older, accounting for up to 20–40%^1^. MPP is usually mild and self-limiting. However, approximately 12% of hospitalized children with MPP will progress to severe *Mycoplasma pneumoniae* pneumonia (SMPP)^2^, which is a serious health risk for children. SMPP may be associated with serious intrapulmonary and extrapulmonary complications. Intrapulmonary complications include plastic bronchitis, pleural effusion, lung consolidation and necrosis, pulmonary embolism, and extrapulmonary complications include myocardial injury, abnormal liver function, kidney damage, anemia and encephalitis^1,3^. Children with SMPP are highly susceptible to life-threatening conditions such as diffuse alveolar hemorrhage, pulmonary embolism and acute respiratory disease syndrome, with lower survival rates and intensive use of medical resources^4^. Therefore, finding independent risk factors for SMPP and further establishing an accurate predictive nomogram have important clinical value for the early identification of SMPP and prevention of disease progression.

Peripheral blood examination is an important method to detect MPP and can be used to find risk predictors of MPP and SMPP^5^. Biomarkers such as white blood cells (WBC), neutrophil percentage (N%), lymphocyte percentage (L%), albumin to globulin ratio (AGR), C-reactive protein (CRP) and erythrocyte sedimentation rate (ESR) can represent the immunoinflammatory response and often correlate with the severity of the disease. In recent years, the mean platelet volume (MPV) and neutrophil-to-lymphocyte ratio (NLR) have also been associated with inflammatory responses and used in the diagnosis and treatment of MPP^6,7^. In addition, studies have shown that a combination of clinical features and biomarkers can help physicians identify MPP early. Clinical features such as age, pleural effusion, fever days, primary disease, and wheezing are potential risk factors for MPP^8,9^. Therefore, we collected data on the above variables for research.

Nomograms are rarely used in MPP risk prediction. Our goal is to develop the world's first dynamic nomogram that accurately predicts SMPP in children. In this study, we retrospectively evaluated the clinical features and representative biomarkers of the MPP described above and identified independent risk factors for SMPP. Finally, we successfully built a dynamic nomogram model with 11 independent risk factors, which showed good calibration and discriminatory power.

## Materials and methods

### Patient data

**Figure 1:**
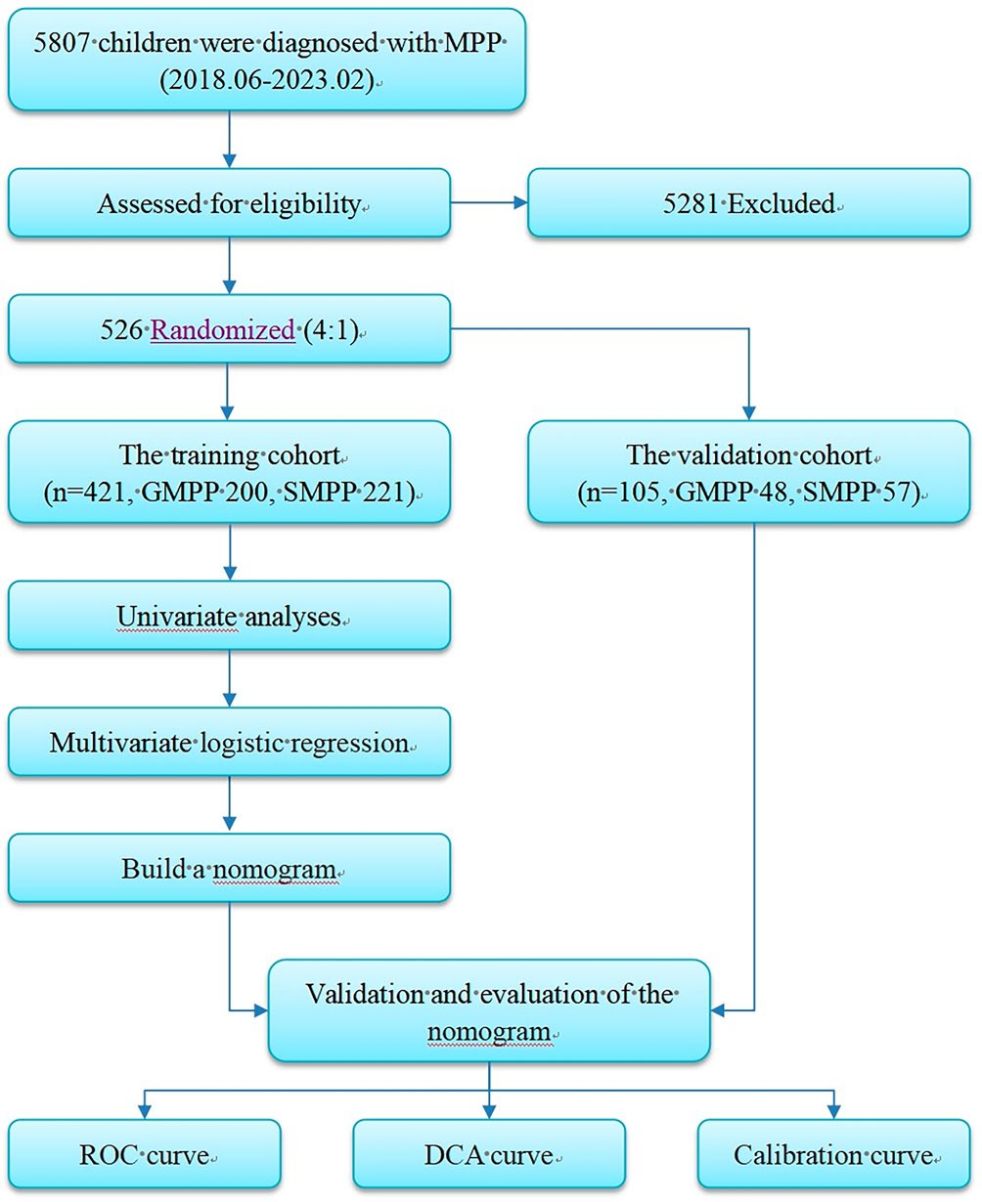
Flow chart of the study process.

The study included 5807 children with MPP in Henan Children's Hospital from June 2018 to February 2023. According to the inclusion and exclusion criteria (Table 1), 5281 children were excluded, and 526 were finally included as study subjects.The children were divided into the general *Mycoplasma pneumoniae* pneumonia (GMPP) group (n = 248) and the SMPP group (n = 278) according to the Guidelines for the Diagnosis and Treatment of Community-Acquired Pneumonia in Children (2019 version)^10^. The grouping criteria are detailed in Table 2. The dataset was randomly split into the training cohort and the validation cohort at a ratio of 4:1. The detailed flow chart is presented in Fig. 1. This study was approved by the Medical Ethics Committee of Henan children’s hospital (2023-K-081). All methods were carried out in accordance with relevant guidelines and regulations. The informed consent was obtained from all subjects and/or their legal guardians.

**Table 1:** Inclusion and exclusion criteria.

Inclusion criteria	The patients were younger than 18 years old
	The patients were diagnosed with MPP according to the Guidelines for Diagnosis and Treatment of Mycoplasma Pneumonae Pneumonia in Children (2023 Edition). Meet the following three points: With fever, cough, wheezing, dyspnea and other respiratory manifestations; Chest imaging examinations were consistent with pneumonia; Compliance with ≥ 1 item: Single serum MP antibody titer ≥ 1:160 (PA method) or double serum MP antibody titers increased by 4 times or more; Positive MP-DNA or MP-RNA
	The clinical data of the patients were complete
	Obtain the informed consent of the children's parents
Exclusion Criteria	The patients had recently received immunotherapy or hormone therapy
	Admission during the MPP recovery period (patients with a disease course of more than 4 weeks, stable temperature for more than 1 week, improvement of chest imaging)

**Table 2:** Grouping criteria.

SMPP group	The patients met the inclusion criteria and had any of the following performances: Poor general situation; Conscious disorder, cyanosis, respiratory dysfunction; Hypoxemia, assisted breathing (groan, flaring of alaenasi, three depressions sign), intermittent apnea, oxygen saturation < 92%; Persistent hyperpyrexia for more than 5 days or ultra-hyperpyrexia; Dehydration or food refusal; Chest imaging showed the following findings: unilateral lung infiltration ≥ 2/3, multilobular lung infiltration, pleural effusion, pneumothorax, atelectasis, lung necrosis, lung abscess; Extrapulmonary complications
GMPP group	The patients met the inclusion criteria and had no above performance

### Data collection

The following clinical data were obtained: (1) representative biomarkers with immunomodulatory and inflammatory effects: WBC, N%, L%, NLR, AGR, CRP, ESR, MPV; (2) clinical characteristics: age, sex, primary disease, coinfection, pleural effusion, fever days and wheeze. Fasting venous blood was collected within 24 h after admission for blood analysis. Chest X-ray or chest CT was performed 3 days before or within 3 days after admission, and the results were recorded.

### Statistical analysis

The dataset was analysed statistically using SPSS 27.0 software and ShinyApp (RStudio, 4.2.1). Quantitative variables were expressed as the mean ± standard deviation ($\overline{\chi }$ ± S) or median and quartile [M (Q1, Q3)], while categorical variables were expressed as the number and percentage (n [%]). *P* < 0.05 was considered statistically significant.

First, univariate analyses (t-test, χ^2^ test, nonparametric test, and univariate logistic regression) were performed on the dataset. Second, multivariate logistic regression was used to identify the independent risk factors for SMPP. Third, we built a dynamic nomogram. Finally, the dynamic nomogram was validated and evaluated. The detailed flow chart is presented in Fig. 1.

### Ethics approval and consent to participate

This study was approved by the Medical Ethics Committee of Henan children’s hospital (2023-K-081). Informed consent was obtained from the parents or guardians of all study participants.

## Results

### Patient characteristics

In this study, 526 eligible subjects, including 294 males (55.89%) and 232 females (44.11%), were randomized into the training cohort and the validation cohort. The results showed no significant difference in these variables between the two cohorts (*P* > 0.05).

In the training cohort, the levels of WBC, N%, NLR, CRP, ESR and MPV in the SMPP group were higher than those in the GMPP group, as were incidence of primary disease, coinfection, pleural effusion, fever days ≥ 7 and wheeze. However, age, L% and AGR were lower in the SMPP group (*P* < 0.05, Table 3).

**Table 3:** Clinical characteristics and biomarkers of the patients.

Variables	All (n = 526)	Training cohort		*p*	Validation cohort		*p*
		GMPP (n = 200)	SMPP (n = 221)		GMPP (n = 48)	SMPP (n = 57)	
Biomarkers							
WBC, 10^9^/L	8.95 (7.03,12.05)	8.59 (6.25,11.69)	9.64 (7.51,12.82)	0.006	8.65 (7.31,10.23)	8.39 (6.32,11.68)	0.622
N%, %	62.50 (48.80,75.50)	59.70 (46.28,69.13)	67.75 (49.88,80.38)	< 0.001	(57.94 ± 15.89)	(63.56 ± 18.29)	0.207
L%, %	29.60 (18.55,41.75)	32.55 (24.28,45.40)	25.30 (15.33,40.15)	< 0.001	34.70 (24.10,39.60)	28.25 (15.73,37.45)	0.069
NLR	2.12 (1.17,4.03)	1.82 (1.00,2.87)	2.68 (1.20,5.20)	< 0.001	1.71 (1.30,2.92)	2.35 (1.45,4.90)	0.067
AGR	1.66 (1.38,2.00)	1.83 (1.57,2.08)	1.51 (1.23,1.87)	< 0.001	(1.86 ± 0.46)	(1.58 ± 0.49)	0.606
CRP, mg/L	6.60 (0.80,23.27)	4.16 (0.50,12.60)	13.45 (2.40,40.13)	< 0.001	5.36 (0.80,23.07)	7.30 (0.80,39.95)	0.330
ESR, mm/h	35.00 (20.00,52.50)	29.00 (18.00,40.50)	40.00 (20.25,61.00)	< 0.001	(34.50 ± 20.89)	(38.54 ± 19.94)	0.721
MPV, fL	9.40 (8.90,10.00)	9.30 (8.88,9.80)	9.60 (9.00,10.30)	0.002	9.10 (8.70,9.70)	9.35 (8.70,10.00)	0.344
Clinical characteristics							
Age, months	52.00 (27.00,83.00)	60.00 (36.00,84.00)	44.00 (21.00,78.75)	0.004	51.00 (33.00,71.00)	43.00 (25.50,84.00)	0.526
Sex	294 (55.89%)			0.503			0.232
Female	232 (44.11%)	84 (42.00%)	100 (45.20%)		25 (52.10%)	23 (40.40%)	
Male	294 (55.89%)	116 (58.00%)	121 (54.80%)		23 (47.90%)	34 (59.60%)	
Primary disease	60 (11.41%)			< 0.001			0.297
No		191 (95.50%)	180 (81.40%)		45 (93.80%)	50 (87.70%)	
Yes		9 (4.50%)	41 (18.60%)		3 (6.30%)	7 (12.30%)	
Coinfection	246 (46.77%)			< 0.001			< 0.001
No		131 (65.50%)	104 (47.10%)		29 (60.40%)	16 (28.10%)	
Yes		69 (34.50%)	117 (52.90%)		19 (39.60%)	41 (71.90%)	
Pleural effusion	146 (27.76%)			< 0.001			0.065
No		158 (79.00%)	141 (63.80%)		41 (85.40%)	40 (70.20%)	
Yes		42 (21.00%)	80 (36.20%)		7 (14.60%)	17 (29.80%)	
Fever days	362 (68.82%)			< 0.001			< 0.001
< 7		91 (45.50%)	39 (17.60%)		24 (50.00%)	10 (17.50%)	
≥ 7		109 (54.50%)	182 (82.40%)		24 (50.00%)	47 (82.50%)	
Wheeze	153 (29.09%)			< 0.001			0.006
No		164 (82.00%)	138 (62.40%)		39 (81.30%)	32 (56.10%)	
Yes		36 (18.00%)	83 (37.60%)		9 (18.80%)	25 (43.90%)	

All patients were tested for etiology, and there was significant difference in coinfection between the two groups (*P* < 0.05, Table 3). Among the study subjects, 246 patients (46.77%) were infected with other pathogens, among which viral coinfection was the highest, followed by bacterial coinfection, and fungal coinfection was the lowest. Coinfection with multiple pathogens was not uncommon. Haemophilus influenzae, respiratory syncytial virus and parainfluenza virus were the top three pathogens with the highest coinfection rate.

Among 526 MPP children, 60 patients (11.41%) have primary diseases. The most common primary disease was congenital heart disease (12 cases), followed by epilepsy (7 cases) and asthma (7 cases). In addition, they may have primary diseases, including hernias, rhinitis and sinusitis, and methylmalonic acidemia.

In addition, there was no statistically significant difference in sex between the SMPP group and the GMPP group (*P* > 0.05, Table 3).

### Selected risk factors for model

First, univariate logistic regression was performed for the above variables with statistically significant differences, and 14 potential risk factors for SMPP were screened out, including WBC, N%, L%, NLR, AGR, CRP, ESR, MPV, age, primary disease, coinfection, pleural effusion, fever days ≥ 7 and wheeze (Table 4).

**Table 4:** Logistic Regressions of SMPP Study.

Variables	Univariate logistic regression			Multivariate logistic regression		
	OR	95% CI	*p*	0R	95% CI	*p*
WBC, 10^9^/L	1.07	(1.02–1.12)	0.004			
N%, %	1.02	(1.01–1.03)	< 0.001			
L%, %	0.98	(0.97–0.99)	< 0.001			
NLR	1.20	(1.11–1.31)	< 0.001	1.15	(1.02–1.29)	0.025
AGR	0.20	(0.12–0.32)	< 0.001	0.42	(0.22–0.79)	0.007
CRP, mg/L	1.03	(1.02–1.04)	< 0.001	1.02	(1.00–1.04)	0.013
ESR, mm/h	1.02	(1.01–1.03)	< 0.001	1.02	(1.00–1.03)	0.013
MPV, fL	1.42	(1.14–1.77)	0.002	1.57	(1.09–2.24)	0.014
Age, months	0.99	(0.99–1.00)	0.008	0.98	(0.97–0.99)	< 0.001
Coinfection	2.14	(1.44–3.17)	< 0.001	2.33	(1.33–4.09)	0.003
Pleural effusion	2.13	(1.38–3.30)	< 0.001	2.07	(1.05–4.10)	0.036
Primary disease	4.83	(2.28–10.23)	< 0.001	3.72	(1.41–9.77)	0.008
Fever days ≥ 7	3.90	(2.50–6.07)	< 0.001	3.45	(1.87–6.38)	< 0.001
Wheeze	2.74	(1.74–4.31)	< 0.001	2.04	(1.07–3.87)	0.030

Then, multicollinearity analysis and multivariate logistic regression were performed for those potential risk factors. The results showed that age, AGR, NLR, CRP, ESR, MPV, coinfection, pleural effusion, primary disease, fever days ≥ 7 and wheeze were independent risk factors for SMPP (*P* < 0.05, Table 4). The AUC, cut-off value, sensitivity and specificity are shown in Table 5.

**Table 5:** The results of the ROC curve.

Variables	AUC	Cut-off value	Youden index	Sensitivity	Specificity	95%CI	*p*
Age, months	0.58	31.50	0.18	0.41	0.77	(0.53–0.64)	0.004
AGR	0.70	1.55	0.34	0.546	0.80	(0.65–0.75)	< 0.001
NLR	0.62	3.12	0.26	0.45	0.80	(0.56–0.67)	< 0.001
CRP, mg/L	0.68	15.49	0.28	0.45	0.82	(0.63–0.73)	< 0.001
ESR, mm/h	0.61	52.50	0.24	0.34	0.90	(0.56–0.67)	< 0.001
MPV, fL	0.59	9.95	0.18	0.37	0.81	(0.53–0.64)	0.002
Coinfection	0.59	–	0.18	0.53	0.66	(0.53–0.64)	0.001
Pleural effusion	0.58	–	0.15	0.36	0.79	(0.52–0.63)	0.007
Primary disease	0.57	–	0.14	0.19	0.96	(0.52–0.63)	0.013
Fever days ≥ 7	0.64	–	0.28	0.82	0.46	(0.59–0.69)	< 0.001
Wheeze	0.60	–	0.20	0.38	0.82	(0.54–0.65)	0.001

### Development and validation of the dynamic nomogram

**Figure 2:**
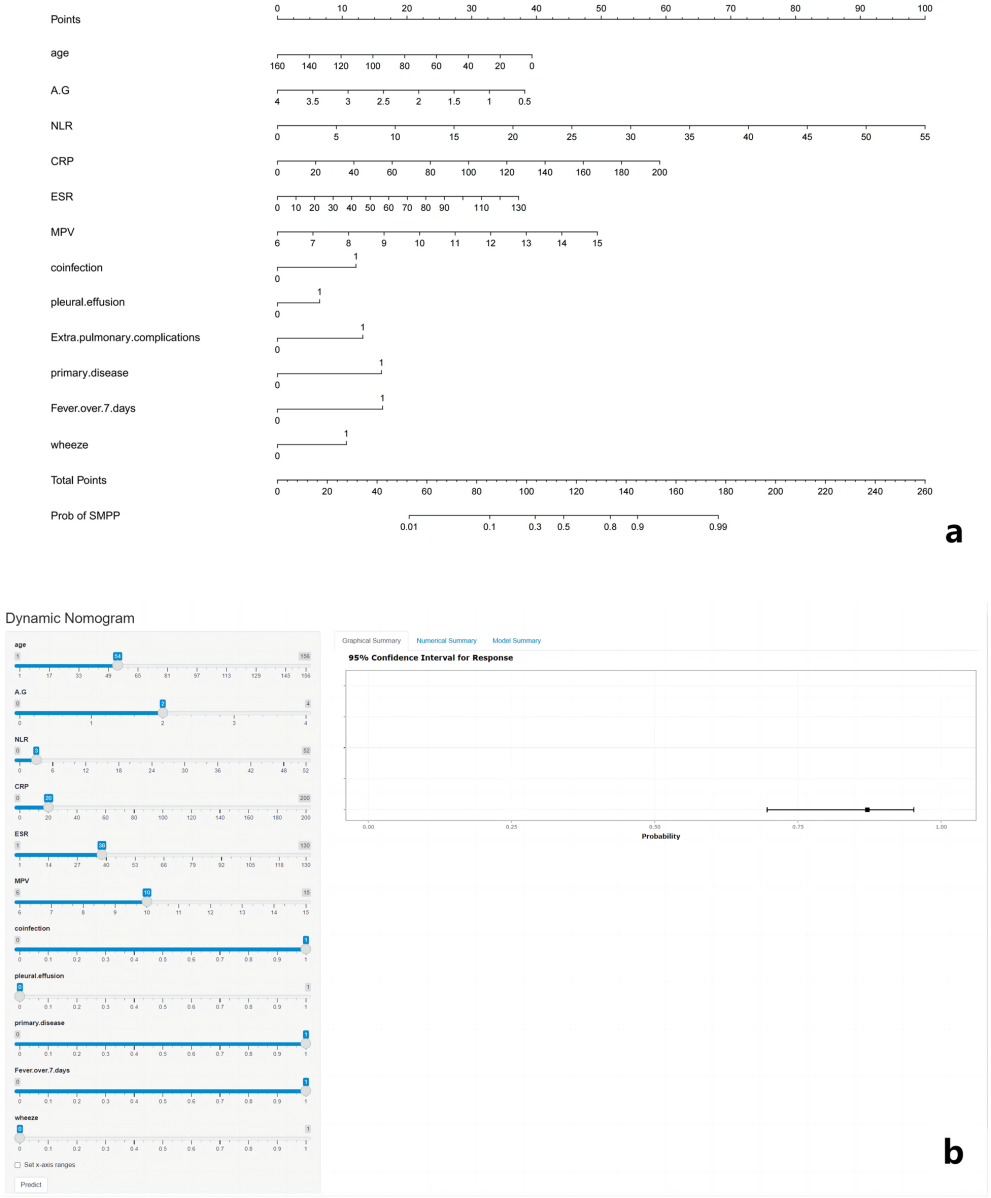
Nomogram prediction model for SMPP diagnosis. (**a**) Established nomogram in the training cohort by incorporating the eleven parameters. (**b**) Online dynamic nomogram (https://ertongyiyuanliexiantu.shinyapps.io/SMPP/).

According to the results of the multivariate logistic regression, we constructed a nomogram model including age, AGR, NLR, CRP, ESR, MPV, coinfection, pleural effusion, primary disease, fever days ≥ 7 and wheeze (Fig. 2). The higher the total score of all risk factors, the higher the risk of SMPP.

**Figure 3:**
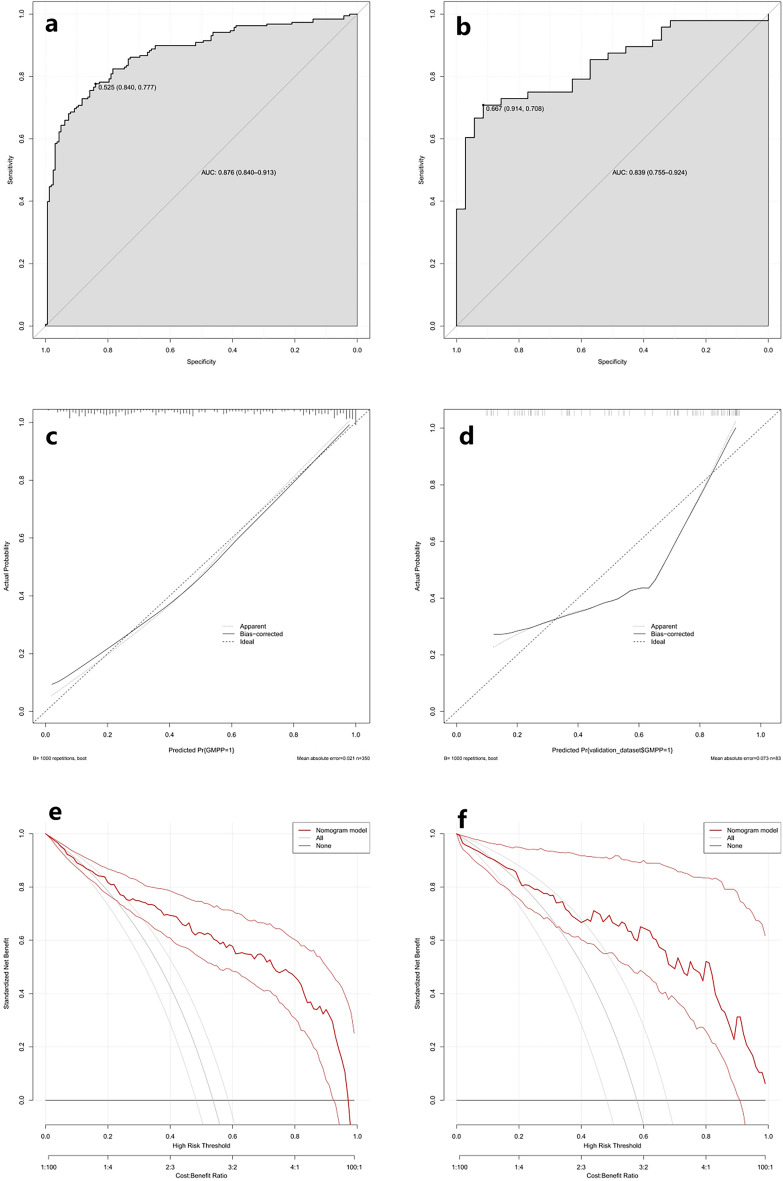
Evaluation of the validity and reliability of the nomogram. The ROC curves of the training cohort (**a**) and the validation cohort (**b**); the calibration curves of the training cohort (**c**) and the validation cohort (**d**); the DCA curves of the training cohort (**e**) and the validation cohort (**f**).

The consistency index (C-index) of the model is 0.87, with good accuracy and discriminability. The ROC curve, DCA curve and calibration curve were used to assess the performance of the nomogram, which all showed that the dynamic nomogram has excellent clinical value (Fig. 3). As shown in Fig. 3, the ROC curves of the model showed good predictive ability, and the AUCs in the training cohort and the validation cohort were 0.867 and 0.840, respectively. The calibration curve of the training cohort was relatively close to the ideal curve, indicating that the predicted results are consistent with the actual results. The DCA curves showed that the nomogram had good benefits for clinical use.

To simplify the application of the model in clinical practice, we also built an online dynamic nomogram (https://ertongyiyuanliexiantu.shinyapps.io/SMPP/). For example, a 54-month-old child with MPP has an 87.1% probability of SMPP, whose characteristics were fever days ≥ 7, AGR of 2, NLR of 3, CRP 20 mg/L, ESR 38 mm/h, MPV of 10 fL, coinfection, primary disease, no pleural effusion, and no wheeze (Fig. 2).

## Discussion

In recent years, *Mycoplasma pneumoniae* (MP) has gradually become one of the main pathogens of community-acquired pneumonia in children, with high mortality and complication rates^11^. Current laboratorial diagnosis methods for MP infection included culture, serological test and various nucleic acid amplification-based assays. Although MP culture is considered to be the "gold standard" for the diagnosis of MP infection, it is difficult to use in clinical diagnosis because of its time consuming. Therefore, serological tests and PCR-based assays (DNA/RNA) were the main tools for the diagnosis of MP infection in clinical practices, and the combination can greatly improve the reliability and accuracy in diagnosis of MP infection^5,12^. Of note, the incidence of SMPP has been on the rise in recent years. Early identification of SMPP is beneficial to rational treatment, reduce sequelae and optimize the utilization of medical resources, which has gradually become the center and key issue in the diagnosis and treatment of MPP.

The logistic regression results of this study showed 11 independent risk factors for SMPP, including (1) representative biomarkers with immunomodulatory and inflammatory effects: AGR, NLR, CRP, ESR, MPV and (2) clinical features: age, coinfection, pleural effusion, primary disease, fever days and wheeze. After the above variables were incorporated into the SMPP risk prediction model, the C-index, the ROC curve, the DCA curve and the calibration curve all showed that the dynamic nomogram has excellent clinical value.

MPP can be seen in all stages of childhood, but it is more common in preschool and school-age children. Kutty^2^ and Xia Wang^13^ noted that the median age of MPP children was 7 (4.0–11.0) years and 5.1 (4.0–7.9) years, respectively. Ding Lin ^14^ further found that SMPP was mainly found in preschool children. Our study showed that the median age was 60 months in the GMPP group and 44 months in the SMPP group, which is basically consistent with the above findings. We also found that age < 31.50 months was an independent risk factor for SMPP in children with MPP, which is thought to be associated with an immature immune system and weaker resistance.

AGR combines serum albumin and globulin, which can be used as a more accurate variable of inflammatory status. Both albumin and globulin play important roles in the body's inflammatory and immune responses^15^. Thus, the AGR can be used as a potential predictor of inflammatory diseases such as MPP. This study innovatively incorporated AGR into SMPP risk prediction. The results showed that AGR was an independent risk factor for SMPP, and the AGR was lower in the SMPP group.

CRP and ESR are recognized variables of inflammation and have been shown in many studies to be significantly elevated in children with MPP and correlated with disease severity. We found that children with CRP > 15.49 mg/L and ESR > 52.50 mm/h had a higher risk of developing SMPP.

In recent years, NLR and MPV have been considered to be correlated with the inflammatory status of the body and used in the diagnosis and treatment of MPP^6,7^. However, their relevant studies in SMPP are rare and need further research.

The NLR is a simple biomarker to assess systemic inflammatory status, taking into account both N and L, and has more clinical predictive value than a single variable. In recent years, studies have found that the NLR is associated with MPP and can be used for the diagnosis, severity assessment and risk prediction of MPP^16^. Zhang^17^ found that the NLR increased in patients with MPP and was closely related to the severity of MPP. In addition, it has been shown that the NLR can be used as a predictor of RMPP^18^ and is important in the differential diagnosis of MPP and bacterial pneumonia^7^. Our results showed that the NLR was significantly higher in the SMPP group than in the GMPP grou and was found to be an independent risk factor for SMPP. Elevated NLR is caused by reduced lymphocyte count and/or elevated neutrophil count, suggesting more impaired lymphocyte function and/or higher levels of inflammation in children with SMPP.

MPV is a variable of platelet function, activity and volume, which may be more sensitive than platelets. It has been shown to be associated with a variety of prethrombotic states and inflammatory diseases, such as respiratory diseases^19,20^. However, studies on MPV in MP infection are rare. Qi^21^ MPV was high in patients with MPP. MP may drive inflammation through MPV, and MPV could be a potential biomarker for predicting MPP or even SMPP^22^. In our study, MPV was higher in the SMPP group than in the GMPP group and could be an independent risk factor for SMPP. We considered that MPV reflects the degree of platelet activation, whose overactivation of platelets can lead to excessive inflammatory responses. Therefore, the higher the MPV elevation is, the more intense the inflammatory response, and the higher the risk of SMPP.

Children with MPP with coinfection, pleural effusion, primary disease, persistent high fever and wheeze are at significantly increased risk of developing severe and critical illness^5^. And relevant literature has suggested that primary disease and coinfection may be potential risk factors for SMPP^23,24^. The existence of pulmonary coinfection is always accompanied by the increase of the severity of the disease and the complexity of treatment, with higher mortality and complications. SMPP caused by mixed pulmonary pathogens can present as poor efficacy of antimicrobial therapy, who required bronchoscopy intervention^23^. Children with primary diseases, including asthma, primary immunodeficiency, and other diseases, are more likely to develop SMPP because of their poor resistance^5^. Our study confirmed that these variables are independent risk factors for SMPP and have significant clinical significance in SMPP risk prediction models.

In previous studies, the nomogram has rarely been used to predict SMPP in children. To our knowledge, this is the first dynamic nomogram developed and validated that can be used to predict the risk of incidence of SMPP in children. The model combines clinical characteristics and biomarkers and showed good predictive accuracy by validation. Moreover, the variables we included were simple and easily accessible, and this model is suitable for clinical promotion. However, our study was retrospective, and subjects may have selective bias. In the future, we need to conduct long-term, multicenter, prospective studies with larger samples to further demonstrate the value of this SMPP prediction model.

## Conclusion

Age, AGR, NLR, CRP, ESR, MPV, coinfection, pleural effusion, primary disease, fever days ≥ 7 and wheeze were independent risk factors for SMPP in children. A dynamic nomogram based on these 11 risk factors showed good predictive accuracy. This dynamic nomogram prediction model could be a powerful tool to help pediatricians make accurate diagnoses and rational treatments.

## Data Availability

The datasets generated and/or analysed during the current study are not publicly available due the hospital’s policies or confidentiality agreements but are available from the corresponding author on reasonable request.
